# 肺段切除术与肺叶切除术术后肺功能保留的差异

**DOI:** 10.3779/j.issn.1009-3419.2019.03.11

**Published:** 2019-03-20

**Authors:** 天一 隋, 傲 刘, 文捷 矫

**Affiliations:** 266071 青岛，青岛大学附属医院 Department of Thoracic Surgery, the Affiliated Hospital of Qingdao University, Qingdao 266071, China

**Keywords:** 肺肿瘤, 肺段切除术, 肺叶切除术, 肺功能, Lung neoplasms, Segmentectomy, Lobectomy, Pulmonary function

## Abstract

近年来肺癌发病率和检出率逐渐升高，肺段切除术被越来越普遍应用于治疗早期非小细胞肺癌。有学者认为肺段切除术相比肺叶切除术更有利于术后肺功能的保留，也有研究得出两种手术方式在术后肺功能保留方面差异不大，本文就肺段切除术与肺叶切除术术后肺功能变化的相关研究作一综述。

## Abstract (en)

In recent years, the incidence and detection rate of lung cancer have gradually increased, and segmentectomy has been increasingly used to treat early non-small cell lung cancer. Some scholars believe that segmentectomy is more conducive to the preservation of postoperative lung function than lobectomy. Some studies have found that the two surgical methods have little difference in postoperative cost retention. This article deals with segmentectomy and lobectomy. A review of related studies on postoperative pulmonary function changes.

肺癌是常见的恶性肿瘤之一^[1]^，其对人体的健康与寿命有着极大的危害。近年来肺癌发病率越来越高，其死亡率更是达到了恶性肿瘤之首^[2]^。对于早期非小细胞肺癌，外科手术切除是有效的治疗手段。目前，肺叶切除加肺门、纵隔淋巴结清扫术仍是早期肺癌的标准手术方式。随着影像学技术的提高，越来越多直径 < 2 cm的早期肺癌被发现^[3]^。有研究^[4-6]^表明，肺段切除术可适用于直径≤2 cm的非小细胞肺癌。虽然在1995年的一项随机性对照实验^[7]^表明，限制性肺部切除术对比肺叶切除术有着更高的局部复发风险，会降低I期肺癌患者术后生存率，但之后有多项研究^[8-12]^证明肺段切除术及肺叶切除术有相同的术后生存率。欧洲呼吸学会以及欧洲胸外科学会均于2009年推荐使用肺段切除术作为肺叶切除术的替代方案。此外，英国胸科学会以及美国胸科的医师学院也分别于2010年及2013年推荐类似治疗方案^[13-15]^。而目前对于早期肺癌的手术方式仍存在争议，基于两种手术方式有着相同的术后生存率，有越来越多的专家倾向于对Ia期非小细胞肺癌行肺段切除术，并认为肺段切除术保留了更多的肺组织，从而术后肺功能保留效果上优于肺叶切除术，但也有学者认为两者在术后肺功能保留方面差别不大。本文就肺段切除术与肺叶切除术术后肺功能变化的研究进展作一综述。

## 1 肺功能的测量指标

肺功能评估主要包括肺容积评估、肺通气功能评估和肺换气功能评估等。肺通气功能检查是肺功能检查中最重要的指标之一，肺容积是指在安静状态下，测定一次呼吸所出现的气量变化，不受时间限制，理论上具有静态解剖学意义，反映肺泡的含气量，包括基础肺容积和基础肺容量，其中，肺活量（vital capacity, VC）是常用的诊断价值较高的指标。肺通气功能指单位时间内随呼吸运动进出肺的气量和流速，包含气流在气道里通过时的流速及其影响因素，常用的评估肺通气功能的指标有用力肺活量（forced vital capacity, FVC）、第1秒用力呼气容积（forced expiratory volume in one second, FEV_1_）、1秒率（FEV_1_/FVC%）、最大自主通气量（maximum voluntary ventilation, MVV）等。肺换气指肺内有效的气体交换，也称“内呼吸”，受通气量和血流量影响，肺弥散功能是肺换气功能的重要部分，肺一氧化碳弥散量（diffusion capacity for carbon monoxide of the lung, DLCO）是常用评估肺弥散功能的指标。也有研究^[16-18]^利用单光子发射计算机断层扫描（single-photon emission computed tomography, SPECT）灌注扫描检查了使用肺段切除术治疗的每个肺叶的肺功能的保留，如Yoshimoto等及Nomori等利用SPECT测量术前、术后患者肺功能的变化。

对术前、术后总体肺功能检查的常用指标有VC、FVC、FEV_1_、MVV、DLCO等，其中，FVC是指最大吸气至肺总量后，以最大气量、最快速度所能呼出的全部气量；FEV_1_是指FVC第1秒内所呼出的气量容积，是肺功能障碍的最主要和最常用的指标。目前多数研究大多用FEV_1_与FVC来评估术后患者的总体肺功能，并计算了术前术后变化的百分比，从而比较两种手术方式对于患者术后肺功能保留方面的差异。

## 2 术后肺功能保留方面的差异

有研究^[19, 20]^认为，术后肺功能在手术后3个月内逐渐恢复，之后保持稳定。张艳娇等^[21]^在2016年对60例胸腔镜肺段切除术组患者及56例胸腔镜肺叶切除术组的患者进行回顾性分析，分别于术前、术后第3天和术后3个月检查测试患者肺功能，胸腔镜肺段切除术与胸腔镜肺叶切除术在FVC及FEV_1_的组间差异均有统计学意义（*P*=0.033; *P*=0.029; *P*=0.019; *P*=0.044），可见肺段切除术较肺叶切除术能更好地保留肺功能。在短期肺功能的比较上，赵纯等^[22]^纳入了122例术后肺癌患者，比较了两种手术方式在术后1个月时FVC、FEV_1_的差异，同样得出胸腔镜下肺段切除术更有利于保留肺功能。

术后患者肺功能的恢复与时间有很大关系^[23-25]^，当患者术后时间超过3个月时，两种手术方式在术后肺功能保留方面差异是否同样明显，Koike等^[8]^在2016年的研究中将术后测量时间延长至术后6个月，纳入了32例肺叶切除术患者及33例限制性切除术患者（30例肺段切除术患者，2例肺部楔形切除术患者及1例术中转为肺叶切除术的患者），对两组手术方式的术中/术前FVC及FEV_1_的比值进行统计学分析，得到限制性切除术更有利于保留术后的肺功能（*P*=0.032, *P*=0.005）。同时Koike等^[8]^对患者的5年、8年的总生存率（overall survival, OS）及无病生存率（disease-free survival, DFS）进行比较，发现两种手术方式在术后生存率方面无明显差异^[8]^。多项研究^[9-12]^也证明了此观点。Saito等^[26]^将短期复查肺功能时间点设定在术后1个月，而长期复查肺功能时间点设定在术后6个月，对126例肺叶切除术后患者及52例肺段切除术后患者进行回顾性分析肺功能测量指标包括VC和FEV_1_，发现两组术后1个月-6个月的FEV_1_指标差别均较大，且手术方式是术后VC或FEV_1_减小的唯一独立相关因素，可见术后6个月内肺段切除术在术后肺功能保留方面要优于肺叶切除术。Harad等^[27]^的研究也证明了在术后6个月时肺段切除术在术后肺功能保留方面更有优势。一些研究^[28, 29]^也得出在术后6个月时，肺段切除术更有利于保留术后肺功能。

以上研究对术后肺功能测量时间间隔较短，为了研究患者术后长期肺功能在不同手术方式间的差异，在一项纳入142例早期非小细胞肺癌术后患者的研究中，当术后12个月时，亚肺叶叶切除术（包括肺段切除术及肺部楔形切除术）更有利于术后肺功能的保留，并且右上叶或右中叶手术在术后肺保留方面优于其他部位^[30]^。朱冰等^[31]^测量了术后1年时两种手术方式在FVC、FEV_1_、MVV 3项指标上的差异，发现胸腔镜下肺段切除术对肺功能的保护作用明显优于肺叶切除术，同时还发现在手术时间、术中出血量、清扫淋巴结的个数、术后住院时间、术后6个月肿瘤标志物水平、术后并发症等指标上两种手术方式并没有太大差异，可见两种手术方式在治疗早期肺癌的疗效均较好。此外，Kim等^[23]^的一项研究中，同时对比了短期及长期术后肺功能的差异，将短期和长期时间点分别定在术后3个月和术后12个月，对患者术前与术后FVC、FEV_1_、DLCO的变化量进行分析比较，发现在短期和长期随访中，亚肺叶切除术组均更好的保留了肺功能。值得注意的是，在随访期间，肺叶切除术组的FVC和DLCO的改善程度明显优于亚肺叶切除术组，而肺叶切除术组和亚肺叶切除术组之间的FEV_1_改善程度无显著差异。作者认为FVC可以通过残余肺的代偿恢复，而FEV_1_是气道阻力的指标，肺叶切除术组未显示FEV_1_的更大改善。更有Kobayashi等^[25]^纳入445例患者，对术后1年时及术后5年时的肺功能进行测量及比较，得出在术后1年-5年间，两种手术方式在肺功能上均有下降，且肺功能的降低是由患者年龄增加引起的，并不受手术方式的影响，同时肺段切除术在术后长期保留有着一定的肺功能优势。但是，在术后长期肺功能保留方面，有的研究得出两种手术方式差异并不明显：Suzuki等^[24]^纳入37例肺段切除术和33例肺叶切除术的患者进行回顾性分析，研究比较了患者术后2个月与7个月-12个月的肺功能变化，结果显示两组术后肺功能仅在术后2个月内存在显著差异。在术后7个月-12个月，肺叶切除术组和肺段切除术组的FVC恢复率分别为93.2%和93.4%（*P*=0.97），肺叶切除术和肺段切除术组的FEV_1_恢复率分别为88.8%和87.8%（*P*=0.82）。在术后随访时间大于13个月与术后7个月-12个月相比，各组肺功能变化无统计学差异。Suzuki等^[24]^认为在术后的早期阶段，肺段切除术比肺叶切除术具有肺功能保留优势。虽然肺段切除术比肺叶切除术可以保留更多的肺实质，从理论上讲，在肺功能保留方面，肺段切除术应该优于肺叶切除术。但数据表明，相比于肺段切除术，肺叶切除术拥有更好的术后残余肺扩张，这可能是导致两种手术方式的术后长期肺功能差别不大。对比之前Kim等的研究，Suzuki等纳入的研究对象为肺段及肺叶切除术的患者，但是Kim等的研究同时纳入了楔形切除术患者，且两者所纳入患者的标准不同，手术切除的部位有所偏差，这些都有可能造成实验结果的差异。所以，我们并不能明确得到两种手术方式在术后肺功能保留方面的长期效果。

在得到两种手术方式在术后肺功能保留方面差异不大的文献中，大多认为是术后残余肺的代偿导致了这一现象，Gu等^[32]^在术后6个月统计了肺段切除术患者及肺叶切除术患者的肺功能，得到两者在FEV_1_及DLCO的变化量上差别并不大，并且Gu等测量了肺叶切除术组和肺段切除术组切除的每个肺段肺功能的平均损失，结果表明在肺段切除术组术后单个肺段的FVC、FEV_1_以及DLCO的损失明显大于肺叶切除术组。Gu等认为这是肺叶切除术后残余肺的扩张优于肺段切除术导致的。Nomori等^[33]^在2016年一项研究中认为肺叶切除术后残余肺组织的代偿能力优于肺段切除术，所以肺段切除术所切除肺组织虽然少于肺叶切除术，但是术后长期的肺功能保留优势并不明显，且两种手术方式下术后患者的生存率无明显差异。随后Nomori等^[18]^利用SPECT测量术后患者整体肺活量、同侧非手术叶肺功能及对侧肺功能，发现肺段切除术比肺叶切除术保留了更多的全肺功能，因为它不仅保留了对侧肺功能，而且同侧非手术叶的肺功能有着更好的代偿能力。同时，测量得到两种手术方式的术后区域肺功能均有增加，Nomori等认为是残余肺代偿所致。

大多数的研究采用了回顾性分析，手术方式有时会受到主刀医生主观判断的影响，并且不同的主刀医生对于肺部解剖及手术的精确度上的差异可能会对术后患者肺功能的恢复造成一定的影响。并且在不同的研究中，对于纳入的病例有着不同的指标，手术切除的部位数量上有所偏差，这些都可能造成不同的研究结果之间出现差异。各实验在术后肺功能测量时间上的跨度过大，无法更加精确的得到具体时间点上肺功能的变化，导致我们只能以术后“短期”及“长期”来描述术后肺功能的差异，并且各研究不同的肺功能测量时间点，也可能是导致实验结论差异的影响因素之一。虽然有的研究得出两种手术方式对于术后肺功能有差异，也有实验得出差异不大，但是在涉及不同时间段测量肺功能的实验中，我们可以看到两种术式在肺功能保留差异上逐渐缩小，这一现象可能是术后残余肺代偿所致。

## 3 影响术后肺功能变化的因素

### 3.1 术前肺功能储备

近年来，有研究认为对于术前肺功能差的患者应该优先选择肺段切除术。在Martin-Ucar等^[34]^的研究中，肺段切除术组与肺叶切除术组的患者术前FEV_1_%预计值分别为44%和45%，术后随访复查肺功能的时间为3个月-6个月，发现当患者术前肺功能差时术后肺段切除术拥有更好的肺功能保留。但是与之相反，Kashiwabara等^[35]^对FEV_1_%预计值在50%-80%的患者进行了分组分析，比较了各组的术后肺功能变化量，得出了对FEV_1_%预计值> 70%的患者，肺段切除术在术后肺功能保留方面优于肺叶切除术，而当FEV_1_%预计值< 70%时，两种手术方式术后肺功能变化差异并不明显。因此，术前肺功能储备有可能会影响患者术后肺功能保留。

### 3.2 手术切除部位

Kim等^[23]^研究比较了同一肺叶行不同手术方式后的肺功能变化，即根据切除肺叶不同及所行手术方式不同分组比较术后肺功能的变化：右肺上叶或右肺中叶、右肺下叶、左肺上叶和左肺下叶。切除的肺叶不同，肺功能的改善是不同的，右肺上叶或右肺中叶的肺叶切除术组显示患者术后FVC和DLCO有显著改善，在术后12个月时，肺叶切除术组与亚肺叶切除组的FVC和DLCO相似。Kim等^[23]^认为在右肺中叶或右肺上叶的肺叶切除术后，右肺下叶的代偿扩张导致的向上移位使右支气管成角增大，导致气道阻力增加，影响FEV_1_的恢复，从而导致右肺上叶及右肺中叶术后FEV_1_无显著改善。可见，手术切除的肺叶影响患者术后肺功能的恢复。

### 3.3 切除肺段数

关于切除的肺段数是否会影响术后肺功能的保留，在一项纳入159例肺部切除术患者的研究^[36]^中，与较大的肺部切除术（切除肺段数为3个-5个肺段）相比，保留肺实质的切除术（切除肺段数为1个-2个肺段）可以有着更好的肺功能保留效果。此外，Yoshimoto等^[16]^利用单光子发射计算机断层扫描灌注扫描检查肺段切除术后的每个肺叶的肺功能保留，发现与切除单个肺段相比，切除多个肺段后的肺功能显著降低。同样的，Nomori等^[33]^研究发现，肺段切除术切除的肺段数≤2个与 > 2个的患者术后FEV_1_变化值分别为3%与10%，肺段切除数量越多，则术后肺功能变化越明显。更有Harada等测量出肺叶切除术平均切除的肺段数量为（3.9±1.1），肺段切除术平均切除肺段数量为（1.9±0.9），且切除的肺段数与术后肺功能丢失量呈正相关，有统计学意义，表明肺段切除术在保留肺功能方面优于肺叶切除术^[27]^。而Hwang等^[37]^对肺段切除术的肺段数进行细分，发现单一肺段切除*vs*两个肺段切除*vs*三个肺段切除的肺段切除术术后FEV_1_变化之比为8.3%:11.7%:13.8%，差别无统计学意义（*P*=0.18）。由此可见，理论上，肺段切除数量越多，患者损失的肺实质越多，术后肺功能保留效果应越差，但术后肺功能保留受多因素影响，肺段切除数量是否独立影响患者术后肺功能仍需进一步研究。

## 4 总结

肺段切除术在术后肺功能保留方面优于肺叶切除术，但是优势随着术后时间的延长而减少，原因可能与术后残余肺的代偿有关。在术后长期方面，两种手术方式在术后肺功能保留方面的差异还不明确。术后复查肺功能的时间多分为长期与短期，时间跨度过大，可能会对研究结果有一定的影响。尽管目前普遍认为肺段切除术更适合用于术前肺功能差及合并较多肺部疾病的患者，但却鲜有纳入术前肺功能较差患者及术前合并各类肺部疾病患者的研究报道。并且手术方式多受术者临床决策的影响，不同的术者在手术切除的精准度上也有区别，这些可能也会影响研究结果。所以关于两种手术方式术后肺功能保留的差异还需要进一步的研究。

影响患者术后肺功能变化的因素主要包括术前肺功能储备、手术切除部位及切除的肺段数等。因此，需要更多大样本、前瞻性、多中心的随机对照研究，减少术后肺功能复查的时间跨度，延长术后肺功能的测量时间点，并对术前肺功能较差及合并各类肺部疾病患者进行分组分析。
